# Screening of cardiovascular risk assessment accuracy of anthropometric indices in Indian children and adolescents

**DOI:** 10.12688/wellcomeopenres.16385.2

**Published:** 2021-05-24

**Authors:** Mohit Aggarwal, Shailendra Singh, Anubhuti Bansal, Bapu Koundinya Desiraju, Anurag Agrawal

**Affiliations:** 1 CSIR-Institute of Genomics and Integrative Biology, New Delhi, Delhi, 110007, India; 2 Academy of Scientific and Innovative Research, Ghaziabad, 201002, India; 3 Translational health science and technology institute, Faridabad, 121001, India

**Keywords:** Growth chart, Body mass index, Waist circumference, Waist to height ratio, Hypertension, Pediatric central adiposity

## Abstract

**Background**: Body mass index (BMI) is the most popular anthropometric marker to define obesity and cardiometabolic risk. BMI is limited in its ability to discriminate central adiposity and other indices such as waist circumference (WC), and waist to height ratio (WHtR) could be a better choice. In this study, we aimed to evaluate the relative accuracy of these indices for the prediction of hypertension in Indian children and young adults.

**Methods**: Anthropometric indices and blood pressure measurements were obtained in 2609 adolescent children and young adults (10-20 years) across a national residential school system. Z-scores were calculated for anthropometric parameters using the Box-Cox-Cole-Green method and World Health Organization (WHO) growth charts. Hypertension was defined using the sex, age and height specific cutoffs for systolic blood pressure. Receiver operator curve (ROC) analysis was performed to examine the predictive ability.

**Results**: Girls had higher BMI for age in our dataset (p < 0.001), along with higher odds for stunting (95% CI: 1.21 – 1.88) as well as central obesity (95% CI: 2.44 – 3.99). Hypertension was seen in 10.6% of the subjects, with higher age, and higher BMI or WHtR as the predictors. Prehypertension was higher in males (p <0.001). WHtR had acceptable but modest discrimination ability for hypertension (AUC > 0.6) in boys (AUC=0.62) and girls (AUC=0.66). Performance of BMI was better in boys (AUC = 0.67) but poor in girls (AUC = 0.55)

**Conclusion**: WHtR was a better predictor of hypertension in Indian adolescent girls and could be used as an augmented parameter to BMI for a better assessment of cardiovascular risk.

## Introduction

Childhood obesity has been linked to an increased risk of type-2 diabetes and cardiovascular diseases in adulthood ^1, 2^. There has been a continual increase in the global prevalence of obesity in children and adolescents in the past decades. Overweight and obesity in children has increased up to 1.5 times between 1980 to 2013 ^3^. There are globally around 38 million preschool children (under age 5 years) and 340 million school children and adolescents (age 5–19 years) that are overweight or obese according to the World Health Organization (WHO) ^4^. India has seen an unprecedented rise in the burden of non-communicable diseases (NCDs) in recent times ^5^. A large proportion of this is due to diseases that have been linked to childhood obesity by studies conducted in high-income countries. Scarcity of such studies in the Indian context along with an over-burdened healthcare system also contribute greatly to the existing burden of NCDs. Effective monitoring in childhood with screening tools that are non-invasive and do not require much clinical expertise may help to overcome this problem to some extent. 

Population growth charts based on anthropometric parameters and indices have been an integral component of the public health system to monitor childhood growth and development. Health care professionals use them as a primary screening tool to assess chronic health conditions that are mostly characterize by an abnormal growth pattern. General practice is to generate age and sex specific growth charts or “normograms” and define statistical cut-points based on population-specific distribution of these markers. There has also been a proposition of having a standard growth recommendations for markers such as BMI for general childhood populations ^6^. Despite World Health Organization’s BMI for age chart for preschool children gaining much wider acceptance, we still mostly rely on ethnicity-based growth charts ^7^. BMI is a good marker of obesity up to the point where there is uniform deposition of fat. Indians and south Asians are generally characterized by a higher abdominal adiposity and lesser muscle mass ^8, 9^. The population specific norms of BMI in these populations may not hold much discriminatory power and need to be augmented with other markers of central adiposity. The best approach is to evaluate Fat Free Mass Index (FFMI) by estimating total body fat. Methods such as Dual-Energy X-ray Absorptiometry (DXA) and Bioelectrical Impedance Analysis (BIA) can measure total body fat and its regional distribution, but are costly and mostly limited to research settings. Measurement of subcutaneous fat through skinfold measurements is affordable, but require much higher examination skills compared to other body measurements. Waist circumference (WC) however is relatively simpler, and a more direct measure of abdominal fat deposition than BMI. WC also requires a sex and age specific normative charts like BMI and therefore both the parameters lack uniformity in their cut-points used to define obesity and are prone to bias. Waist to height ratio (WHtR) seems to be a more robust marker of central adiposity whose variations are independent of age and sex. A simple cut-off of 0.5 has been suggested for WHtR that can used to define obesity in most ethnic groups ^10^. So far, there have been no studies that have determined the prognostic value of these markers in the Indian population on a national scale

In this study we aimed to examine the accuracy of these 3 anthropometric markers in the prediction of hypertension in a population of school children and adolescents from low-middle socioeconomic background. The primary question that we asked was whether WC and WHtR hold any additional prognostic value that can be used along with the BMI for the better definition of overweight and obese.

## Methods

### Study participants and setting

School children aged 9–20 years were enrolled from 14 districts studying in residential schools (Jawahar Navodaya Vidyalaya) of an Indian government scheme (Navodaya Vidyalaya Samiti), to form a cohort named SOLID (Study Of Lung function and Its Development). Study sites were selected in a manner that it would account for most of the geographical, cultural and ethnical diversity of the country. A List of 13 states and union territories was suggested to the Navodaya Vidyalaya Samiti (NVS), who randomly approved 1 study site in each of these regions except for Rajasthan, where we were approved with one additional site (Jaisalmer) situated in the Thar desert. Southern (Bengaluru, Thiruvananthapuram and Puducherry) and north-eastern India (Phodong, Ribhoi and 24 N Pargana) were represented by 3 sites each. Leh and Mandi represented high altitude and hilly regions, respectively. Remaining sites provided a good representation of north and central India. Children in these boarding schools mostly belong to the lower middle socioeconomic strata and stay in a similar living arrangement for most part of the year. Since children in these schools have an unrestricted access to good nutrition along with a regime of regular physical training, it technically provides an environment where the undernourishment and physical inactivity could have a lesser role than general population. Cross-sectional screenings of 2609 participants were performed from 2017–2019. Students of class 6 ^th^, 9 ^th^ and 12 ^th^ were enrolled for the study. Participants were excluded if they had any chronic diseases or felt unwell on the day of measurement. A sample size of 2500 had been targeted for gender specific evaluation of environmental exposure related differences in spirometry values, to detect changes of 100 ml or more with 80% power at 5% significance level, assuming exposure rate of 25%. While this study was designed for measuring lung function differences, this is generalizable to other parameters for an effect size of about 0.2 standard deviations. For the present study, the margin of error is less than 2.5% at 95% confidence level, given a 10–20% prevalence for a minimum of 1000 subjects for each sex.

### Ethics and consent

Study was approved by the institutional human ethical committee of CSIR-Institute of genomics and integrative biology (Ref No: CSIR-IGIB/IHEC/2017-18). Written parental consent and verbal subject assent were taken for all the study participants. Parental information sheet and consent forms were distributed for students of class 6 ^th^, 9 ^th^ and 12 ^th^ in the selected schools by NVS prior to school visits. School’s nursing staff obtained written consent from parents and answered their questions regarding the study and its scope. Study participants were had the study explained to them and asked for their verbal assent on the day of measurement.

### Anthropometric assessments and z-scores

Standing height, weight and waist circumference was measured in all the participants. A digital weighing scale (Omron) and stadiometer (IS Indosurgicals) were used for the measurement of weight in kilograms (Kg) and height in centimeters (cm) respectively. Waist circumference in centimeters were measured using a flexible, non-stretchable vinyl tap measure. Body mass index (BMI) was calculated as weight in Kg per unit square of standing height in cm. Same equipments were used for anthropometry throughout the study, in the supervision of same set of examiners. Data was recorded in the digital sheet on site. Outlier correction was performed to remove possible erroneous data entries.

Z-scores for BMI for age (zbfa) and height for age (zhfa) were calculated using the World Health Organization (WHO) growth chart to evaluate the developmental standing of study participants on the global scale ^11^. WHO defines z-scores below -2 for zbfa and zhfa as “thinness” and “stunting” respectively, calculated from the 2007 growth standards. Z-scores were also evaluated from the present data using the Box-Cox-Cole-Green method, for BMI for age and waist for age, to examine their predictive ability in the screening of hypertension. 50 ^th^ percentile of BMI for age obtained was compared with the growth charts of WHO and Indian academy of pediatrics (IAP) ^12^.

### Blood pressure measurement and definition of hypertension

Systolic and diastolic blood pressures were evaluated on a digital measuring device (Omron HEM 7120) in sitting position once after a rest of about 5 minutes. Subjects were classified into groups: “Normal”, “Prehypertension”, “Stage 1 Hypertension”, and “Stage 2 Hypertension”, based on the cutoffs for systolic blood pressure from National Heart, Lung, and Blood Institute ^13^. Low blood pressure in children may be normal or reflect mild dehydration in hot conditions, it was therefore not included as a classification or recorded as part of the study.

### Statistical analyses

Kruskal-Wallis one-way analysis of variance was performed to compare the 50 ^th^ centile of BMI amongst growth charts of IAP, WHO and the present study. Chi-square test of independence was performed to examine the association of sex with the prevalence of stunting, central obesity and hypertension. Odds ratios were calculated to quantify the effect of sex on outcomes such as central obesity and stunting. Independent t-test was used for the comparison of means between two groups.

Age and sex specific normograms were generated for BMI and waist circumference by Box-Cox-Cole-Green (BCCG) method using “ refcurve 0.4.2” software and “ gamlss” package (version 5.1.7) ^14, 15^. “refcurve 0.4.2” was used for the model selection and centile plots, and “gamlss” for the calculation of z-scores and distributional parameters. Outliers were removed from the training data for BMI (outliers were removed for height and weight) and waist circumference using the Interquartile range (IQR) method. Values were omitted if they were 1.5 times IQR below the first quartile or above the 3 ^rd^ quartile. Best model was selected with the lowest Bayesian information criterion (BIC) for the given set of expected degrees of freedom (edf) values for median (mu=M), coefficient of variance (sigma=S) and Box-Cox parameter (lambda=L, implemented as nu in “gamlss”). L, M and S are measures of skewness, central tendency and dispersion, respectively

The receiver operator characteristic (ROC) method was used to calculate the area under ROC curve (AUC) using the “ pROC” package (version 1.16.2) ^16^. Ideal cutoff for the predictor was calculated using the “closest.topleft” argument which defines the cut-point as the point closest to the point (0,1). The Delong test was used for the comparison of predictive ability (AUCs) amongst the three anthropometric indices. R (version 3.6.1) was used for all the statistical analyses and data visualization.

## Results

### Study participants description

**Figure 1.**
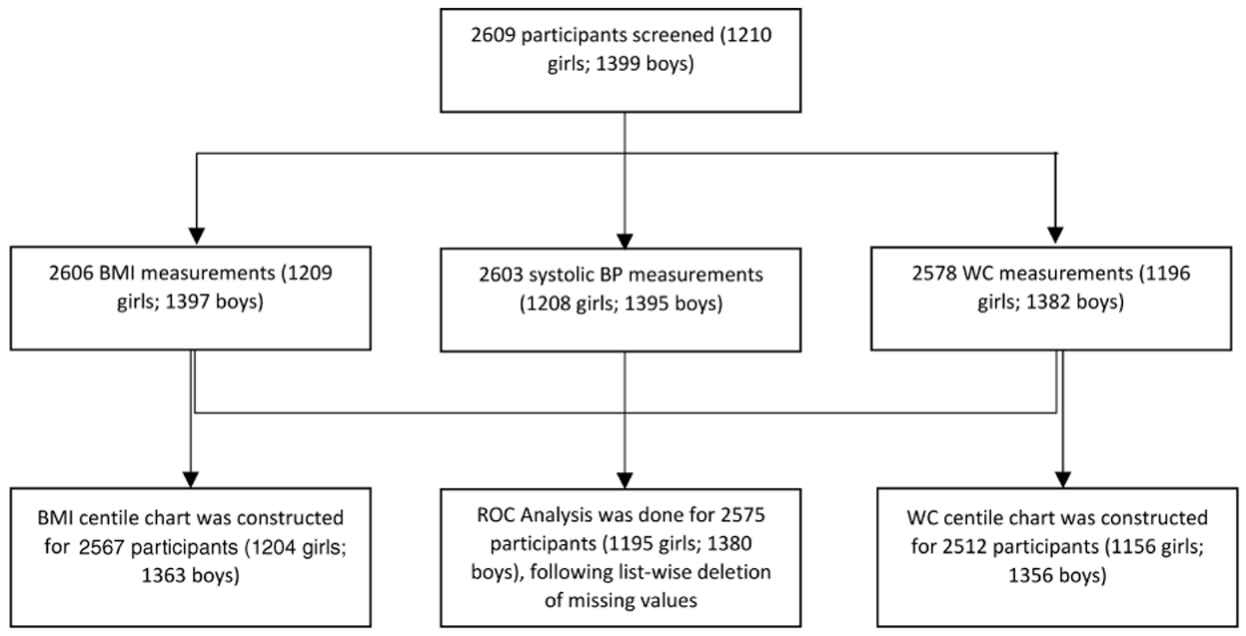
Study Profile. BMI: Body-Mass Index, BP: Blood Pressure, WC: Waist Circumference, ROC: Receiver Operator Characteristics.

**Figure 2.**
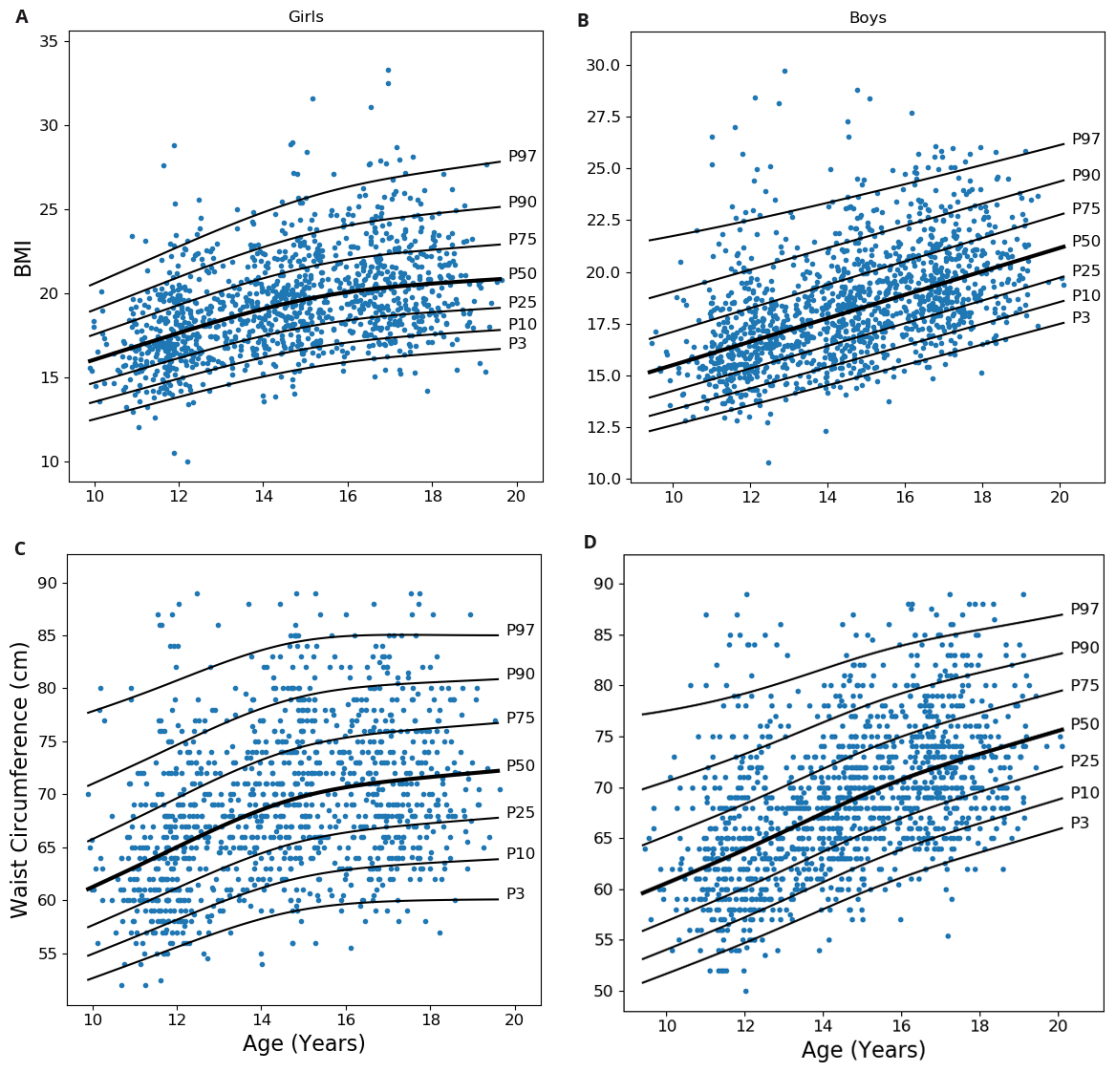
Centile curves of body mass index and waist circumference. Centile plot generated using “Box-Cox-Cole-Green” method showing smoothed curves for 3, 10, 25, 50, 75, 90 and 97 percentiles. **A** and **B** are showing the centile curve for BMI for age in 1204 girls and 1363 boys, respectively. **C** and **D** are showing the curve for waist circumference for age in 1156 girls and 1356 boys, respectively. BMI: Body mass index.

2609 subjects (1210 girls, 1399 boys) were screened for anthropometric parameters and blood pressure measurements from 14 districts ( Figure 1 and Table 1 ^17^). BMI, WC and systolic pressure was missing for 3, 31 and 6 participants, respectively. List wise deletion was done for the multivariate analysis (ROC) by omitting participants having missing values for any of the concerned parameters: BMI, WC, WHtR and systolic pressure. Z-scores of BMI for age (zbfa(WHO)) and height for age (zhfa(WHO)) was calculated for 2579 subjects (1199 girls, 1380 boys) aged 9–19 years using the WHO growth chart. Z-score for weight for age could not be calculated as it was unavailable for children above the age 10 years and for the sake of uniformity it was left out from the analysis, instead of calculating it manually from some Indian reference equation. Data for 2567 and 2512 participants was used, following outlier correction, to construct centile charts for BMI and WC, respectively ( Figure 2). Z-scores of BMI for age (zbfa) and waist for age (zwfa) were calculated using these charts, which were further used as predictors in the ROC analysis. Table 2 and Table 3 ^17^ summarize the age specific distribution parameters (L: Box-cox parameter, M: Median and S: coefficient of variance) of BMI and WC in girls and boys. ROC analysis was done for 2575 participants (1195 girls, 1380 boys) for whom values were available for all the three anthropometric indices, zbfa, zwfa and WHtR. Using the National heart, lung and blood Institute cut-offs for systolic pressure, prevalence of hypertension was calculated in 2603 participants (1208 girls, 1395 boys) between age 9–20 years. 

**Table 1.** Study population description. Data was collected from 14 districts in between 2017–2019.

	Girls						Boys					
District	N	Age (Years)	Height (cm)	Weight (Kg)	BMI (Kg/m ^2^)	Waist Circumference (cm)	N	Age (Years)	Height (cm)	Weight (Kg)	BMI (Kg/ m ^2^)	Waist Circumference (cm)
Bengaluru	90	14.5 (4.05)	151 (10.6)	43.5 (13)	18.8 (4.44)	69 (13.2)	131	14.7 (4.45)	159 (22.1)	47 (19.8)	18.2 (4.08)	68 (12)
Bharatpur	61	14.3 (4.84)	151 (8.5)	42 (15)	18.2 (4.49)	64 (8.5)	101	13.4 (3.95)	157 (17.8)	42 (15.5)	17.1 (2.83)	65 (7.62)
Chandigarh	86	14.4 (4.23)	150 (9.45)	42 (10)	18.4 (3.6)	66 (7)	100	14.4 (4.93)	159 (20.8)	42 (19.8)	17.5 (3.78)	66 (10.2)
Gumla	100	15.8 (3.94)	151 (6.55)	44 (6)	19.2 (3.17)	71.8 (9)	103	16.2 (3.39)	160 (8.3)	48 (10.5)	18.5 (2.24)	69 (6.5)
Jaisalmer	55	14 (5.49)	154 (10.2)	44 (14)	18.2 (4.37)	71 (11)	144	14.6 (4.32)	162 (21.6)	46.5 (19)	17.6 (3.37)	71 (9)
Jhajjar	68	14.5 (3.35)	151 (12)	43 (10)	18.7 (3.87)	64.8 (6.12)	121	14.4 (4.11)	158 (15.6)	44 (16)	18 (3.04)	68 (10.2)
24 N Pargana	76	14.8 (3.98)	153 (7.55)	43 (11)	18.6 (3.95)	66.5 (12)	95	14.5 (4.15)	160 (15.1)	48 (18)	18.5 (4.63)	72 (12.7)
Leh	111	14.4 (3.62)	154 (7.9)	47 (10.8)	19.8 (3.24)	70 (7)	61	14.3 (4.16)	158 (15.3)	47 (17)	18.4 (3.1)	69 (8)
Mandi	89	14.5 (4.36)	152 (9.7)	44 (14)	18.1 (4.25)	68 (12)	107	14.8 (4.32)	161 (18.1)	46 (16)	17.6 (3.5)	68 (11)
Phodong	100	14.7 (5.53)	148 (10.5)	45 (11.2)	19.7 (4.41)	76.5 (9.25)	64	17.2 (4.11)	161 (15.3)	51 (18.2)	19.5 (3.75)	72 (7.25)
Puducherry	119	15.1 (4.26)	150 (7.6)	42 (12.5)	18.7 (4.32)	69 (11.5)	80	15.3 (2.56)	161 (19.8)	49 (19)	18 (3.7)	67 (10.4)
Puri	70	14.6 (3.38)	150 (8.4)	46 (15.8)	19.9 (5.42)	70 (12)	121	14.6 (3.92)	160 (12.8)	51 (15)	20 (4.95)	70 (13)
Ribhoi	94	15.5 (3.5)	148 (7.88)	42 (8)	19.5 (3.48)	65.5 (9.97)	76	15.7 (3.54)	157 (12.7)	46 (12)	18.8 (2.62)	66.7 (6.5)
Thiruvananthapuram	91	14.5 (3.17)	152 (11)	46 (13)	19.5 (3.99)	68 (10.2)	95	14.8 (5.08)	160 (23)	50 (21.5)	18.6 (4.27)	69 (14)

Data is n, median (IQR).

**Table 2.** Age -specific distribution parameters of BMI and WC for Girls between age 9–20. Summary of the age specific first three moments of the distribution (L, M and S), obtained using Box-Cole-Green-Method, of BMI and waist circumference. Data from 1204 and 1156 subjects were used for BMI and waist circumference, respectively. BMI: Body mass index, L (lambda): Box-cox parameter, M (mu): median, S(sigma): coefficient of variance.

	BMI (Kg/m ^2^)			Waist circumference (cm)		
Age (Years)	L	M	S	L	M	S
10	0.0232	16	0.132	-2.39	61.2	0.097
10.5	-0.0315	16.4	0.132	-2.23	62.2	0.0967
11	-0.0861	16.8	0.132	-2.07	63.1	0.0964
11.5	-0.141	17.2	0.133	-1.91	64.1	0.0961
12	-0.195	17.6	0.133	-1.75	65	0.0959
12.5	-0.25	18	0.133	-1.59	66	0.0956
13	-0.305	18.4	0.133	-1.43	66.9	0.0953
13.5	-0.359	18.7	0.133	-1.27	67.7	0.0951
14	-0.414	19.1	0.133	-1.11	68.5	0.0948
14.5	-0.469	19.4	0.133	-0.945	69.2	0.0945
15	-0.523	19.6	0.133	-0.784	69.8	0.0942
15.5	-0.578	19.9	0.133	-0.623	70.3	0.094
16	-0.633	20.1	0.133	-0.463	70.7	0.0937
16.5	-0.687	20.2	0.133	-0.302	71	0.0934
17	-0.742	20.4	0.133	-0.141	71.2	0.0932
17.5	-0.797	20.5	0.133	0.0197	71.4	0.0929
18	-0.851	20.6	0.133	0.18	71.6	0.0926
18.5	-0.906	20.7	0.133	0.341	71.8	0.0924
19	-0.961	20.7	0.133	0.502	72	0.0921
19.5	-1.02	20.8	0.133	0.663	72.2	0.0918
20	-1.07	20.9	0.133	0.823	72.4	0.0916

**Table 3.** Age -specific distribution parameters of BMI and WC for Boys between age 9–20. Summary of the age specific first three moments of the distribution (L, M and S), obtained using Box-Cole-Green-Method, of BMI and waist circumference. Data from 1363 and 1356 subjects were used for BMI and waist circumference, respectively. BMI: Body mass index, L (lambda): Box-cox parameter, M (mu): median, S(sigma): coefficient of variance.

	BMI (Kg/m ^2^)			Waist Circumference (cm)		
Age (Years)	L	M	S	L	M	S
10	-1.83	15.5	0.134	-2.21	60.6	0.101
10.5	-1.76	15.8	0.133	-2.11	61.4	0.0995
11	-1.7	16.1	0.131	-2	62.2	0.0979
11.5	-1.63	16.3	0.13	-1.9	63	0.0964
12	-1.57	16.6	0.128	-1.8	63.9	0.0949
12.5	-1.5	16.9	0.127	-1.7	64.7	0.0934
13	-1.43	17.2	0.125	-1.59	65.6	0.0919
13.5	-1.37	17.5	0.124	-1.49	66.5	0.0905
14	-1.3	17.8	0.122	-1.39	67.4	0.089
14.5	-1.23	18	0.121	-1.28	68.3	0.0876
15	-1.17	18.3	0.119	-1.18	69.2	0.0862
15.5	-1.1	18.6	0.118	-1.08	70	0.0849
16	-1.04	18.9	0.117	-0.973	70.7	0.0835
16.5	-0.971	19.2	0.115	-0.87	71.4	0.0822
17	-0.905	19.5	0.114	-0.766	72.1	0.0809
17.5	-0.838	19.7	0.113	-0.663	72.7	0.0797
18	-0.772	20	0.111	-0.56	73.3	0.0784
18.5	-0.706	20.3	0.11	-0.457	73.8	0.0772
19	-0.64	20.6	0.109	-0.354	74.4	0.076
19.5	-0.574	20.9	0.108	-0.25	75	0.0748
20	-0.508	21.2	0.106	-0.147	75.5	0.0736

### Sex-wise differences in body composition

**Figure 3.**
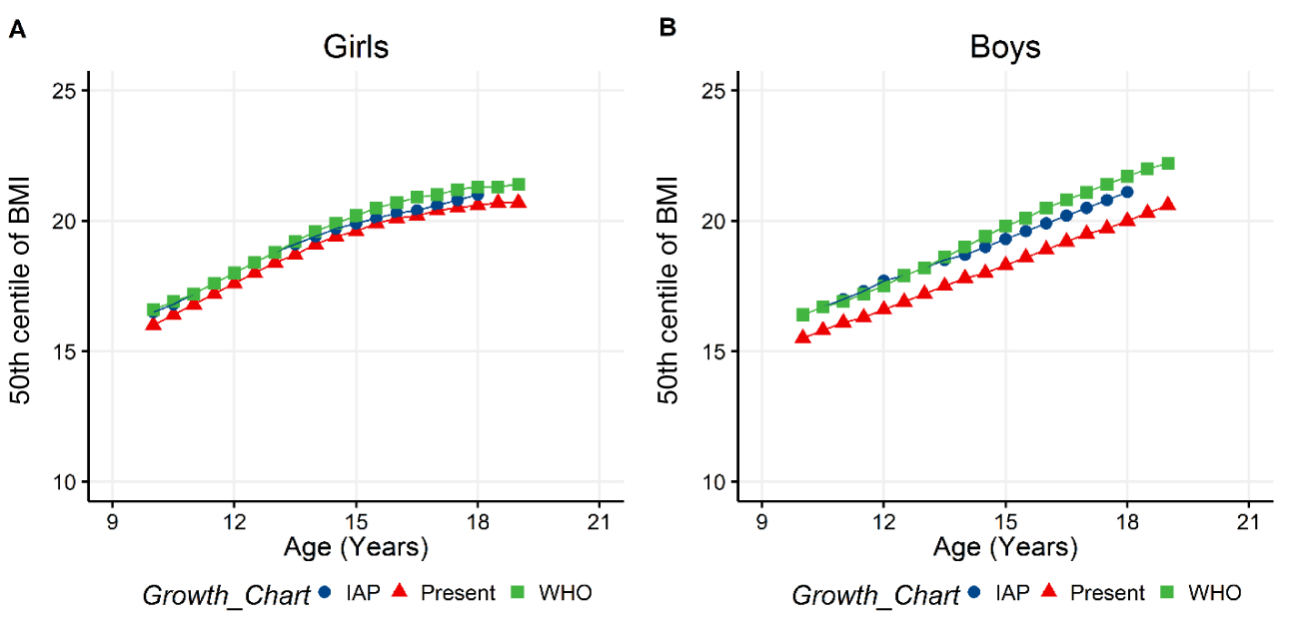
Comparison of 50 ^th^ centiles of IAP, WHO and the present study BMI growth charts. **A** and **B** are showing the comparison of 50th centiles of BMI for age in girls and boys, respectively. 50 ^th^ centile values for present study was obtained using the Box-Cox-Cole-Green method and compared with the growth charts of IAP and WHO. BMI: Body mass index, IAP: Indian academy of pediatrics, WHO: World health organization.

Z-scores of BMI for age (zbfa(WHO)) was higher in girls (girls= -0.24 , boys= -0.56, p < 0.001 ), whereas Z-Height for age (zhfa(WHO)) was higher in boys (girls= -1.13, boys= -0.85, p < 0.001 ). 50 ^th^ percentile of BMI of the IAP and WHO growth charts were observed to not be significantly different to the 50 ^th^ percentile of BMI obtained in the present data using the BCCG method ( Figure 3). Prevalence of thinness was 3.76% and 10% respectively in girls and boys, who had zbfa (WHO) lower than -2. Prevalence of both stunting (zhfa (WHO) < -2) and central obesity (WHtR > 0.5) was significantly different between girls and boys ( Table 4 ^17^). Girls had higher odds of being stunted (OR= 1.5, CI= 1.21 – 1.88) and having central obesity (OR= 3.1, CI= 2.44 – 3.99).

**Table 4.** Prevalence of hypertension, stunting and central obesity in the study population. Prevalence of Prehypertension, HTN-1 and HTN-2 was observed in 1208 girls and 1395 boys. Prevalence of stunting and thinness was observed in 1199 girls and 1380 boys, and central obesity in 1196 girls and 1382 boys. P-values were calculated using chi-square test of independence. P: P-value, HTN-1: Stage 1 hypertension, HTN-2: Stage 2 hypertension, zhfa: Z-score of height for age, WHtR: Waist to height ratio, zbfa: Z-score of Body mass index for age, zhfa and zbfa were calculated using World health organization’s growth chart.

Classification	Girls	Boys	P
Prehypertension	141 (11.67%)	296 (21.2%)	< 0.001
HTN-1	129 (10.68%)	120 (8.6%)	0.44
HTN-2	12 (0.99%)	14 (1%)	0.9
Stunting: zhfa (WHO) < -2	205 (17.10%)	166 (12.03%)	< 0.001
Central Obesity: WHtR > 0.5	240 (20.07%)	103 (7.45%)	< 0.001
Thinness: zbfa (WHO) < -2	45 (3.75%)	138 (10%)	< 0.001

Data is n (%) or p-value.

### Prevalence of hypertension

Stage 2 hypertension (HTN-2) was very infrequent in both the sexes (~ 1%). Prevalence of stage 1 hypertension (HTN-1) was similar in both the sexes ( Table 4 ^17^). Prehypertension was, however, twice as prevalent in boys compared to girls. Overall prevalence of hypertension and prehypertension was 10.6% and 16.8%, respectively. Subjects with hypertension were significantly older (p<0.001), and had a higher BMI (zfba(WHO)) (p<0.001) and WHtR (p<0.001) compared to subjects with normal systolic blood pressure.

### Predictive ability of anthropometric indices for cardiovascular risk

**Figure 4.**
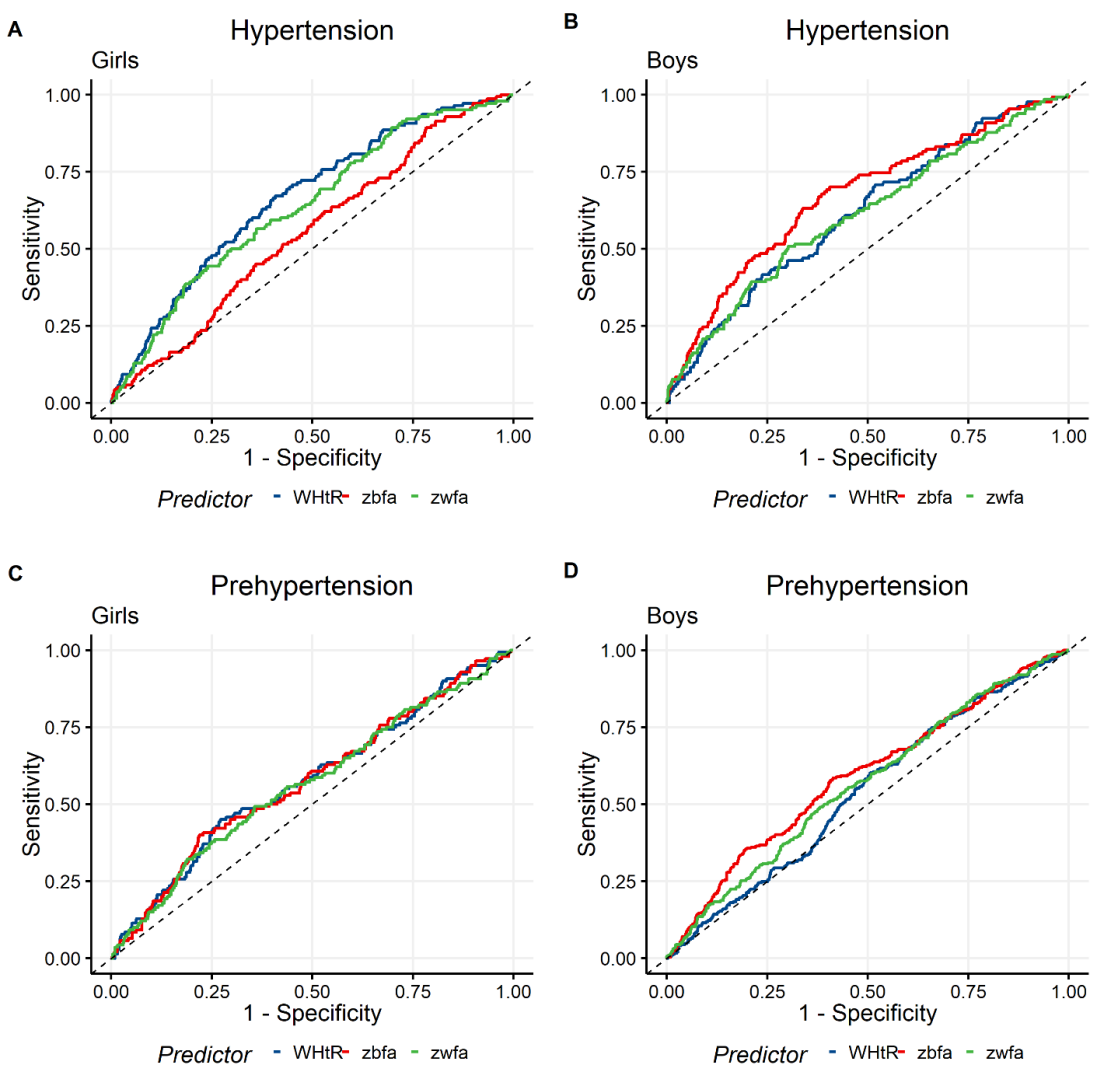
Comparison of AUCs of three anthropometric indices for the screening of hypertension and prehypertension. **A** and **B** are showing the AUCs for the prediction of hypertension in 1055 girls (915 Normal, 128 HTN-1 and 12 HTN-2) and 1086 (956 Normal, 117 HTN-1 and 13 HTN-2) boys respectively, using zbfa, zwfa and WHtR as the predictors. **C** and **D** are the ROC curves for the prediction of prehypertension in 1055 (915 Normal, 140 Prehypertension) girls and 1250 (956 Normal, 294 Prehypertension) boys, respectively. ROC: Receiver operator characteristic, AUC: Area under receiver operator characteristic curve, zbfa: Z-score of body mass index for age, zwfa: Z-score of waist circumference for age, WHtR: waist to height ratio, HTN-1: Stage 1 Hypertension, HTN-2: Stage 2 Hypertension.

AUCs for all the three anthropometric indices (zbfa, zwfa and WHtR) were consistently poor for prehypertension ( Figure 4 and Table 5 ^17^). An improvement in performance was observed for all the indices in the case of hypertension, except for BMI in girls. Girls, who were characterized by higher central obesity, showed a significantly poor AUC for BMI in the prediction of hypertension. WHtR and WC were shown to be the better predictors here with WHtR being slightly more accurate than WC (p=0.02). In boys, BMI was clearly a better predictor of hypertension over the waist circumference (p= 0.005) and WHtR (p=0.02), relating well to the lower prevalence of central obesity and higher prevalence of thinness in this group.

**Table 5.** AUCs, optimal cut-off and validity parameters of anthropometric indices in predicting hypertension. ROC analysis for hypertension (both stage 1 and 2) included 1055 girls (915 Normal, 128 HTN-1 and 12 HTN-2) and 1086 boys (956 Normal, 117 HTN-1 and 13 HTN-2). Prehypertension included 1055 girls (915 Normal, 140 Prehypertension) and 1250 boys (956 Normal, 294 Prehypertension). zfba and zwfa were calculated from the data itself by “BCCG” method. Labels 0 and 1 were used for control and disease, respectively. Hypertension- 0: Normal, 1: HTN-1/HTN-2; Prehypertension- 0: Normal, 1: Prehypertension. ROC: Receiver operator characteristic, AUC: Area under receiver operator characteristic curve, CI: confidence interval, zbfa: Z-score of body mass index for age, zwfa: Z-score of waist circumference for age, WHtR: waist to height ratio, HTN-1: Stage 1 Hypertension, HTN-2: Stage 2 Hypertension, BCCG: Box-Cox-Cole-Green.

Variables	Girls			Boys		
	zbfa	zwfa	WHtR	zbfa	zwfa	WHtR
**Hypertension**						
AUC (95% CI)	0.55 (0.5-0.6)	0.64 (0.59-0.68)	0.66 (0.62-0.71)	0.67 (0.62-0.72)	0.61 (0.56-0.66)	0.62 (0.57-0.67)
Cutoff	0.12	0.29	0.46	0.29	0.41	0.44
Sensitivity	51.43%	56.43%	67.14%	63.08%	50.77%	60.77%
Specificity	57.38%	63.93%	59.23%	66.21%	69.87%	55.75%
**Prehypertension**						
AUC (95% CI)	0.58 (0.52-0.63)	0.57 (0.51-0.62)	0.58 (0.52-0.63)	0.59 (0.55-0.63)	0.56 (0.53-0.6)	0.54 (0.5-0.57)
Cutoff	0.4	0.3	0.47	0.09	0.03	0.43
Sensitivity	45.71%	49.29%	48.57%	58.50%	55.44%	60.20%
Specificity	69.29%	64.37%	67.65%	58.68%	54.92%	49.48%

## Discussion

Low-middle income countries (LMIC) like India, that are in the midst of an epidemiological transition, have a double burden of both a pre-existing prevalence of malnourishment, and a rise in the incidences of obesity owing to nutritional transition and urbanization ^18, 19^. The 2020 Global Nutrition Report stated that up to 10-fold and 5-fold difference exists between the richest to poorest countries in the prevalence of underweight and overweight respectively ^20^. Poor countries had higher prevalence of undernourishment and richer countries had more obesity. India sits somewhere in the middle on an average scale but given the huge nation-wide disparity in the socioeconomic profiles it is possible to identify both the extremes ^21^. We do see a significant prevalence of both central adiposity and stunting in our study as well, sometimes existing together in the same group. For example, both stunting and increased waist to height ratio was higher in girls compared to boys. However, the overall prevalence of stunting in the present study was still much lower than the last reported national average ^22^. This could be attributed to the diet these children receive during their time at the school. The children arrive in these schools aged about 10 years and stay for next 7–8 years, during which they are all provided highly standardized housing, nutrition, sports and education. Therefore, the calorie intake of these children improves significantly. However, Indian diet is majorly vegetarian in origin and the ones who consume animal products, frequency is much less than most other countries. The resultant carbohydrate rich diet, though cures malnourishment, apparently results in increased prevalence of obesity and other related ailments ^23, 24^. This could also be seen in the present study, where rare occurrence of thinness in girls was also accompanied by much higher prevalence of central obesity. We also did not observe any significant differences in the anthropometry of subjects claimed to be non-vegetarians. Though average protein intake was not directly assessed in the present study, similar anthropometry amongst the subjects with different dietary origins indicate to similarity in their macronutrient intake.

We found an unexpectedly high prevalence of pre-hypertension and hypertension in this cohort, with low obesity and high levels of physical activity. Combining pre-hypertension and hypertension together, almost one-third of boys and one-fourth of girls were affected. These were single measurements and are not clinical diagnoses. Within these limitations, we do see expected associations with gender, age and anthropometry. Predictive accuracy of anthropometric markers was poor for pre-hypertension but acceptable in the case of hypertension. Waist to height ratio, as a marker of central adiposity, was superior to BMI or waist circumference alone in girls, but not boys. Given that central adiposity was more common in girls, this is not surprising. Some studies have documented similar results in other ethnic groups where waist measurements were the better correlates of cardiometabolic risks ^25, 26^.

To the best of our knowledge, this is the first dataset from India that includes this much diversity in terms of geography, climate, culture and sub-ethnicity and collected as part of a single study using the same strategy. Our study included school children primarily from lower-middle class and its results are not expected to be extrapolatable to the affluent urban Indian population. The low-middle income group is a very large section of Indian society and their pan-nation representation in study sample is one the major strength of this study. For example, the BMI for age in our data, especially in otherwise healthy and active boys, is somewhat lower than expected from normative curves published for India – possibly from a different socio-economic stratum. Such differences are important to know.

To summarize, we observed that BMI alone as a screening tool for cardiovascular risk fails in populations that are characterized by higher prevalence of central obesity. We therefore suggest it should be used along with other anthropometric indices which are more direct markers of abdominal adiposity, such as WHtR and WC. WHtR may prove to be a better choice here since it does not require age or sex specific norms and is less prone to population bias.

## Data availability

### Underlying data

Figshare: SOLID Study (Anthropometry and Blood Pressure). https://doi.org/10.6084/m9.figshare.13150844 ^17^

This project contains the following underlying data:

- SOLID_anthropometry.csv (Anthropometry and BP data)

Data are available under the terms of the Creative Commons Attribution 4.0 International license (CC-BY 4.0).
