# Supplementary material for: Screening of cardiovascular risk assessment accuracy of anthropometric indices in Indian children and adolescents
Source: Wellcome Open Res. 2021 May 24;5:273. Originally published 2020 Nov 19. [Version 2] doi: 10.12688/wellcomeopenres.16385.2 (PMC10209623; doi:10.12688/wellcomeopenres.16385.2)
Supplement: Peer review history

# Peer review: Reviewer response for version 2

**DOI:** 10.21956/wellcomeopenres.18628.r57700

**Published:** 2023-05-24

**Authors:** Gundu Hirisave Rama Rao

**Affiliations:** 1 Laboratory Medicine and Pathology, Institute for Engineering Medicine, Lillehei Heart Institute, University of Minnesota Twin Cities, Minneapolis, Minnesota, USA

Three decades ago, Dr Bela Shah of Indian Council of Medical Research (ICMR) and Paul M McKeigue of London School of Hygiene and Tropical Medicine, studied South Asian populations in London, and reported that South Asian group had a higher prevalence of diabetes, higher blood pressure, higher plasma triglyceride and lower HDL cholesterol. Based on their findings, they concluded, that within this ethnic group, waist-hip ratio was correlated with glucose intolerance, insulin resistance, triglyceride and associated with a pronounced tendency to central abdominal obesity ^1^. According to Aaron Kelly of University of Minnesota, who has two decades of experience in conducting clinical trials in the treatment of pediatric obesity, this metabolic abnormality leads to endothelial dysfunction and progression of metabolic diseases such as hypertension, excess weight, diabetes, and vascular diseases. His studies have demonstrated that even as short as eight weeks of exercise,- improved fitness, HDL cholesterol and endothelial function ^2^. Compared to the US., the waist-weight ratios are significantly higher in men and women from Chennai, India. Based on their findings the authors concluded that South Asian Indians are particularly predisposed toward central adiposity ^3^. In a later study, Bajaj and associates demonstrated the importance of waist circumference (WC) measurements for mortality, Diabetes and CVD risk prediction and suggested that obesity specific interventions targeting WC in addition to traditional risk factor management, may favorably impact these outcomes ^4^. Excess adiposity is a key risk factor for many chronic metabolic diseases.

 Since the time Framingham Heart Group described risk factors for the development cardiovascular diseases, body mass index (BMI) has been the gold standard and popular marker to define obesity index. In recent years, there are suggestions to use lower BMI cutoffs compared to standard BMI cutoffs to reduce the risk of obesity-related co-morbidities. Kali and associates from Kazakhstan claim to be the first group to explore new BMI and WC cut-offs in this ethnic population. These authors concluded that for central Asia populations, the current recommended BMI and WC cutoffs may not be suitable and further work is needed to establish specific cut-offs for this population ^5^. Patel and associates questioned as to whether the “South Asian Phenotype” is unique to South Asians? They compared the cardiometabolic risk factors in the CARRS study (representing Chennai, New Delhi, , India, Karachi, Pakistan, and the United States: Asians, Blacks, Hispanics, and Whites). They concluded that individuals with “South Asian Phenotype” were 5 to 9 times more likely to exhibit dysglycemia and dyslipidemia in the ‘healthy’ BMI range compared with any other race/ethnic group ^6^.

 In this study Dr Anurag Agrawal and associates studied the anthropometric indices and blood pressure measurements in young children and adolescents, to develop predictive ability of these biomarkers. In the revised version of their manuscript the authors have clarified the methodology, justified the reliability and validity of using the three anthropometric measurements used for collecting the data for these studies. Various methodologies used for collecting the data and the analysis are established standard methods. The study demonstrated the prevalence of prehypertension in boys compared to girls. These studies also demonstrate that the predictability for all the three anthropometric indices were consistently poor for prehypertension. This study also demonstrated the beneficial effect of diet that these children received in the school. It is worth noting, that these researchers found unexpected high prevalence of pre-hypertension and hypertension in this cohort. These findings warrant further investigations with a larger cohort. Their conclusion that BMI alone as a screening tool for cardiovascular risk fails in populations that are characterized by higher prevalence of central abdominal obesity, reminds us of a seminal observation made by Yajnik in his article “Y-Y paradox” ^7^, which demonstrated the limitations of BMI as a measure of adiposity across populations.

 Based on their findings they could have suggested the use of this methodology to recommend screening prehypertensive children and adolescents.

Is the work clearly and accurately presented and does it cite the current literature?

Yes

If applicable, is the statistical analysis and its interpretation appropriate?

Yes

Are all the source data underlying the results available to ensure full reproducibility?

Yes

Is the study design appropriate and is the work technically sound?

Yes

Are the conclusions drawn adequately supported by the results?

Yes

Are sufficient details of methods and analysis provided to allow replication by others?

Yes

Reviewer Expertise:

Thrombosis Research, Platelet, morphology, physiology and pharmacology. Cardiometabolic diseases. Founder, CEO South Asian Society on Atherosclerosis and Thrombosis (www.sasat.org). Emeritus Professor. Laboratory Medicine and Pathology, University of Minnesota

I confirm that I have read this submission and believe that I have an appropriate level of expertise to confirm that it is of an acceptable scientific standard.

## References

[1] : Relation of central obesity and insulin resistance with high diabetes prevalence and cardiovascular risk in South Asians. *The Lancet* .1991;337(8738) : 10.1016/0140-6736(91)91164-P 382-386 10.1016/0140-6736(91)91164-P 1671422

[2] : Inflammation, insulin, and endothelial function in overweight children and adolescents: the role of exercise. *J Pediatr* .2004;145(6) : 10.1016/j.jpeds.2004.08.004 731-6 10.1016/j.jpeds.2004.08.004 15580192

[3] : Comparison of relative waist circumference between Asian Indian and US adults. *J Obes* .2014;2014: 10.1155/2014/461956 461956 10.1155/2014/461956 25328687PMC4189975

[4] : Clinical utility of waist circumference in predicting all-cause mortality in a preventive cardiology clinic population: a PreCIS Database Study. *Obesity (Silver Spring)* .2009;17(8) : 10.1038/oby.2009.44 1615-20 10.1038/oby.2009.44 19265793

[5] : Proposing new body mass index and waist circumference cut-offs based on cardiometabolic risks for a Central Asia population: A feasibility study. *Front Endocrinol (Lausanne)* .2022;13: 10.3389/fendo.2022.963352 963352 10.3389/fendo.2022.963352 36093117PMC9453809

[6] : Is the "South Asian Phenotype" Unique to South Asians?: Comparing Cardiometabolic Risk Factors in the CARRS and NHANES Studies. *Glob Heart* .2016;11(1) : 10.1016/j.gheart.2015.12.010 89-96.e3 10.1016/j.gheart.2015.12.010 27102026PMC4841915

[7] : The Y-Y paradox. *The Lancet* .2004;363(9403) : 10.1016/S0140-6736(03)15269-5 10.1016/S0140-6736(03)15269-5 14726172

# Peer review: Reviewer response for version 1

**DOI:** 10.21956/wellcomeopenres.18024.r42934

**Published:** 2021-03-15

**Authors:** Kotsedi Daniel Monyeki

**Affiliations:** 1 Department of Physiology and Environmental Health, University of Limpopo, Polokwane, South Africa

1. Reliability and validity of anthropometric measurements are not adequately covered.
2. Definition of thinness, stunting could interest some readers.
3. In the statistical analysis it is not clear how the odds ratios were applied. For example use of confounders and or effect modifiers not well articulated.
4. The sample size used in the study is confusing. 
5. Explanation of the results could interest the some readers given the significant number of thinness, central obesity and obesity among these subjects.
6. One wonders if dietary information for this subjects was available for analysis.

Is the work clearly and accurately presented and does it cite the current literature?

Yes

If applicable, is the statistical analysis and its interpretation appropriate?

Partly

Are all the source data underlying the results available to ensure full reproducibility?

Yes

Is the study design appropriate and is the work technically sound?

Yes

Are the conclusions drawn adequately supported by the results?

No

Are sufficient details of methods and analysis provided to allow replication by others?

No

Reviewer Expertise:

Public Health and epidemiology

I confirm that I have read this submission and believe that I have an appropriate level of expertise to confirm that it is of an acceptable scientific standard, however I have significant reservations, as outlined above.

# Response

**Published:** 2021-04-30

**Authors:** Anurag Agrawal

**Affiliations:** CSIR Institute of Genomics and Integrative Biology, India

Thanks. Each of the comment has been addressed individually, please see below.

  

1. **Reliability and validity of anthropometric measurements are not adequately covered.**

**Response/action:**

Reliability of the anthropometric measurements in present Study

 Following text is added in the Methods (Anthropometric assessments and z-scores):

*Same equipments were used for anthropometry throughout the study, in the supervision of same set of examiners. Data was recorded in the digital sheet on site. Outlier correction was performed to remove possible erroneous data entries.*

Validity of anthropometric measurements as marker of adiposity

 Following text is added in the Introduction:

*The population specific norms of BMI in these populations may not hold much discriminatory power and need to be augmented with other markers of central adiposity. The best approach is to evaluate Fat Free Mass Index (FFMI) by estimating total body fat. Methods such as Dual-Energy X-ray Absorptiometry (DXA) and Bioelectrical Impedance Analysis (BIA) can measure total body fat and its regional distribution, but are costly and mostly limited to research settings. Measurement of subcutaneous fat through skinfold measurements is affordable, but require much higher examination skills compared to other body measurements. Waist circumference (WC) however is relatively simpler, and a more direct measure of abdominal fat deposition than BMI.*

  

1. **Definition of thinness, stunting could interest some readers.**

**Response/action: **Following text is added in the Methods (Anthropometric assessments and z-scores):

*WHO defines z-scores below -2 for zbfa and zhfa as “thinness” and “stunting” respectively, calculated from the 2007 growth standards.*

  

1. **In the statistical analysis it is not clear how the odds ratios were applied. For example, use of confounders and or effect modifiers not well articulated.**

**Response/action: **Odds ratios were calculated considering sex as the exposure separately for central obesity and stunting.

 Case I: Exposure (sex): Boys/Girls, Outcomes (central obesity): Normal/Obese

 Case II: Exposure (sex): Boys/Girls, Outcomes (stunting): Normal/Stunted

 Following text is added in the Methods (Statistical analyses):

*Odds ratios were calculated to quantify the effect of sex on outcomes such as central obesity and stunting.*

  

1. **The sample size used in the study is confusing.**

**Response/action: **Margin of error was calculated for the present study for minimum of 1000 subjects for each sex using the following formula.

 Margin of error = Z Ö{p(1-p)/n}

 Z = Z-score

 p = sample proportion

 n = sample size

For p = 10%

 Margin of error = 1.96 Ö{0.1(1-0.1)/1000}

                           = 0.01859419 = 1.86 %

For p = 20%

 Margin of error = 1.96 Ö{0.2(1-0.2)/1000}

                           = 0.02479226 = 2.48 %

 Following text is added in the Methods (Study participants and settings)

*In the present study, the margin of error is less than 2.5 % at 95% confidence level, given a 10-20 % prevalence for minimum 1000 subjects for each sex.*

  

1. **Explanation of the results could interest some of the readers given the significant numbers of thinness, central obesity and obesity among these subjects.**

**Response/action: **Following text is added in the Discussion:

*For example, both stunting and increased waist to height ratio was higher in girls compared to boys. However, the overall prevalence of stunting in the present study was still much lower than the last reported national average* ^1^ *. This could be attributed to the diet these children receive during their time at the school. The children arrive in these schools aged about 10 years and stay for next 7-8 years, during which they are all provided highly standardized housing, nutrition, sports and education. Therefore, the calorie intake of these children improves significantly. However, Indian diet is majorly vegetarian in origin and the ones who consume animal products, frequency is much less than most other countries. The resultant carbohydrate rich diet, though cures malnourishment, apparently results in increased prevalence of obesity and other related ailments* ^2,3^ *. This could also be seen in the present study, where rare occurrence of thinness in girls was also accompanied by much higher prevalence of central obesity.*

  

1. **One wonders if dietary information for these subjects were available for analysis.**

**Response/action: **All study participants belonged to the residential schools of the same governing body. Quality of nutrition was similar in all these schools along with a common physical activity routine. Subjects were asked about their diet type (Vegetarian/Non-vegetarian) and how frequently they skipped meals (Rarely/Often). Anthropometric variables (zbfa, zhfa and WHtR) were not significantly different (Kruskal-Wallis test) amongst the subjects with different diet profiles.

 Following text is added in the Results:

*We also did not observe any significant differences in the anthropometry of subjects claimed to be non-vegetarians. Though average protein intake was not directly assessed in the present study, similar anthropometry amongst the subjects with different dietary origins indicate to similarity in their macronutrient intake.*

**References**

 1          Bhargava M, Bhargava A, Ghate SD, Rao RSP. Nutritional status of Indian adolescents (15-19 years) from National Family Health Surveys 3 and 4: Revised estimates using WHO 2007 Growth reference. *PLoS ONE* 2020; **15**: e0234570.

 2          Sharma M, Kishore A, Roy D, Joshi K. A comparison of the Indian diet with the EAT-Lancet reference diet. *BMC Public Health* 2020; **20**: 812.

 3          Mohan V, Unnikrishnan R, Shobana S, Malavika M, Anjana RM, Sudha V. Are excess carbohydrates the main link to diabetes & its complications in Asians? *Indian Journal of Medical Research* 2018; **148**: 531.

# Peer review: Reviewer response for version 1

**DOI:** 10.21956/wellcomeopenres.18024.r42201

**Published:** 2021-02-12

**Authors:** Vishal Bansal

**Affiliations:** 1 Department of Physiology, Vallabhbhai Patel Chest Institute, University of Delhi, New Delhi, Delhi, India

- Authors have evaluated anthropometric parameters for screening of cardiovascular risk assessment in Indian children and adolescents which is a clinically relevant topic.
- They have rightly pointed out that BMI (Body Mass Index) alone does not provide objective measure as a screening tool.
- Better parameter to assess is FFMI i.e. Fat Free Mass Index instead of BMI, which can be measured by doing Body Composition Analysis through Bio-impedance. This method is reliable, valid and more objective. This method also shows segmental distribution of fat ^1^.
- Both Waist Circumference and Waist to Height Ration (WHtR) has a moderate acceptability as per the results for cardiovascular risk predictability which is quite convincing and can be used as an instant observation in a clinical setting.

Is the work clearly and accurately presented and does it cite the current literature?

Yes

If applicable, is the statistical analysis and its interpretation appropriate?

Yes

Are all the source data underlying the results available to ensure full reproducibility?

Yes

Is the study design appropriate and is the work technically sound?

Yes

Are the conclusions drawn adequately supported by the results?

Yes

Are sufficient details of methods and analysis provided to allow replication by others?

Yes

Reviewer Expertise:

Cardiopulmonary rehabilitation and physiology

I confirm that I have read this submission and believe that I have an appropriate level of expertise to confirm that it is of an acceptable scientific standard.

## References

[1] : Body fat distribution and its association with cardiovascular risk factors in adolescent Iranian girls. *Iran J Pediatr* .2012;22(2) :197-204 23056886PMC3446063
